# Revealing the cellular localization of STAT1 during the cell cycle by super-resolution imaging

**DOI:** 10.1038/srep09045

**Published:** 2015-03-12

**Authors:** Jing Gao, Feng Wang, Yanhou Liu, Mingjun Cai, Haijiao Xu, Junguang Jiang, Hongda Wang

**Affiliations:** 1 State Key Laboratory of Electroanalytical Chemistry, Changchun Institute of Applied Chemistry, Chinese Academy of Sciences, Changchun, Jilin, China; 2 Graduate University of Chinese Academy of Sciences, Beijing, China; 3 Institute of Immunology, The First Bethune Hospital Academy of Translational Medicine, Jilin University, Changchun, Jilin, China

## Abstract

Signal transducers and activators of transcription (STATs) can transduce cytokine signals and regulate gene expression. The cellular localization and nuclear trafficking of STAT1, a representative of the STAT family with multiple transcriptional functions, is tightly related with transcription process, which usually happens in the interphase of the cell cycle. However, these priority questions regarding STAT1 distribution and localization at the different cell-cycle stages remain unclear. By using direct stochastic optical reconstruction microscopy (dSTORM), we found that the nuclear expression level of STAT1 increased gradually as the cell cycle carried out, especially after EGF stimulation. Furthermore, STAT1 formed clusters in the whole cell during the cell cycle, with the size and the number of clusters also increasing significantly from G1 to G2 phase, suggesting that transcription and other cell-cycle related activities can promote STAT1 to form more and larger clusters for fast response to signals. Our work reveals that the cellular localization and clustering distribution of STAT1 are associated with the cell cycle, and further provides an insight into the mechanism of cell-cycle regulated STAT1 signal transduction.

Signal transducers and activators of transcription (STATs) are latent transcription factors that become activated by tyrosine phosphorylation and this modification alters their conformation to form dimers, translocate to the cell nucleus and specifically bind DNA elements^1^,^2^,^3^. They are classically viewed as transducing high-fidelity signals for the cytokine family of ligands and receptors from the plasma membrane to the nucleus, which build a link between cell surface receptors and specific gene expression in the nucleus^4^. The central role of STATs is activating transcription and regulating gene expression, and they function in many physiological activities, such as development, immunity, survival and proliferation^5^,^6^. Their malfunctions are linked to the onset of a wide array of diseases, including developmental disorders and cancers^7^.

The STAT family is comprised of seven distinct members (STAT1-STAT6), among which are the two related forms of STAT5 (STAT5A and STAT5B)^8^. They respond to specific extracellular stimuli and induce the transcription of genes that can invoke different biological functions^4^. Because the STAT family members share similar structural arrangement of their functional motifs^3^, knowledge learned from one member can generally be applied to other family members. Therefore, we mainly focused on STAT1 in this study. STAT1 was first identified as a component of the DNA-binding complex that is activated in response to IFNs^9^,^10^. Alternatively, it can be phosphorylated by growth-factor binding to receptor tyrosine kinases, such as epidermal growth factor (EGF)^11^,^12^ and platelet-derived growth factor (PDGF)^13^.

As STATs are very important transcription factors, their cellular localization, nuclear trafficking and other activities are tightly related with the transcription process. Moreover, the occurrence of transcription directly links to a series of physiological events of cells, which constitute the so-called cell cycle. Previous studies hinted that there might be a relationship between STAT activities and cell cycles. For example, researchers found that STAT1 was essential for the cell growth suppression by inducing CDK inhibitors in response to cytokines^14^, or interacted directly with the G1 cell cycle regulatory cyclin D1 and CDK4^15^. Therefore, accurate cellular localization and nuclear trafficking during the cell cycle plays an integral role in the effective function of STAT proteins. Localizing STAT1 in a cell cycle might provide a solution for therapeutic intervention in diseases that are related with STAT activity and helpful for generating synthetic molecules that function as transcription factors^16^.

Early studies about STAT structures and signaling pathway generally used crystallology, biochemistry, immunofluorescence and so on^17^,^18^,^19^,^20^. However, these methods mostly aimed at the average results of ensemble measurements from many molecules. Conventional fluorescence microscopy has only provided the single-cell or subcellular level of information due to the diffraction limit of light (~200 nm). Fortunately, the recent development of super-resolution fluorescence microscopy has overcome this resolution barrier, generating images with lateral resolutions in the tens of nanometers range^21^,^22^. Among these successful methods, localization microscopy^23^, such as stochastic optical reconstruction microscopy (STORM)^24^,^25^ and photoactivated localization microscopy (PALM)^26^, based on the precise localization of single molecules, has been widely used. It has been proved as a powerful tool particularly suitable for investigating the detailed features of protein distribution at the single molecule level^27^,^28^,^29^. The major principle of this approach is separating the overlap fluorescence signals by only allowing one molecule per diffraction-limited area to fluoresce at a given time. As activated fluorescent molecules are sparsely distributed each time, it is possible to accurately record their coordinates. By obtaining a series of images until molecules bleach, this localization process is repeated and finally a complete fluorescence image is built up based on the identified locations of every detected molecule.

Herein, we utilize direct STORM (dSTORM), which achieves the precision localization by using small molecule dyes that can be switched between fluorescence on/off states, to investigate the localization and nuclear trafficking of STAT1 at the different cell-cycle stage. Due to the improvement of the spatial resolution, we can characterize the distribution of STAT1 proteins in both cytoplasm and nucleus during the cell cycle. The quantitative analyses indicate that the amount of STAT1 in the nucleus is associated with the activity of different cell-cycle stages. Meanwhile, we find that STAT1 proteins mostly exist in the form of clusters especially with cytokine stimulation leading to the significant increase of cluster size and number, which may facilitate faster transduction of signals and activation of transcription.

## Results

### Super-resolution imaging of STAT1 distribution at the different cell-cycle stage

**Figure 1:**
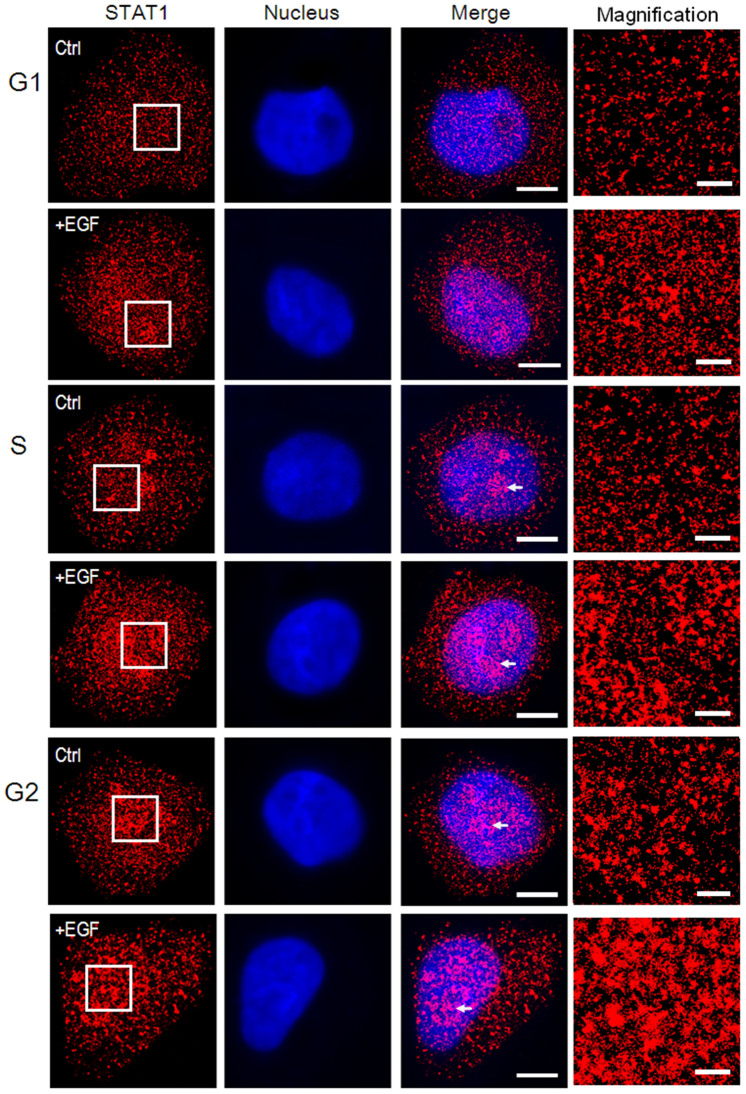
STAT1 expression gradual enhance from G1 to G2 phase and EGF-induced STAT1 transportation into the nucleus. STAT1 labeled with anti-STAT1 antibody and the nucleus labeled with Hoechst33342 in fixed and permeabilized Hela cells were imaged at the different cell-cycle stage with or without EGF stimulation. The first column displays the reconstructed dSTORM images of STAT1 in the whole cell and STAT1 expression increased by degrees from G1 to G2 phase either in control groups or in EGF-induced groups. The second column are the single fluorescent images of the corresponding nucleus. The third column are the merging images of STAT1 and nucleus, which show marked accumulation of STAT1 in the nucleoplasm after EGF stimulation at all cell-cycle stages. Especially in S and G2 phase, STAT1 form many clusters in the nucleus which white arrows point to. The last column shows the boxed areas in the first column (white lines) at higher magnification. Scale bars are 10 μm in the third column and 2 μm in the last column.

Hela cells were firstly synchronized by TdR and collected at different time points for detecting the cell cycle by flow cytometry. The results showed a good synchronization (Supplementary Fig. S1), which ensured the accuracy of sampling each time. To observe the STAT1 localization accurately, we firstly optimized fixation and permeabilization conditions of immunostaining^30^ for dSTORM imaging (Supplementary Fig. S2). There were no significant differences in STAT1 localization between different methods, except that the number of localizations in methanol permeated cells was much less than that in Triton X-100 treated cells. To eliminate the effect of protein extraction or redistribution, the STAT1 localization in living Hela cells transfected with GFP-fused STAT1 plasmids was completed^31^ (Supplementary Fig. S3). Comparing the living cell fluorescence imaging and immunofluorescence, we found that both STAT1 localization and distribution features (e.g. fluorescent spots) were similar except the number and size of fluorescent spots due to the different spatial resolution of two imaging techniques. Therefore, we preferred to use 4% paraformaldehyde fixation and 0.2% Triton X-100 permeablization for immunostaining (see Methods for details). The fluorescence images of STAT1 from 3-D, TIRF and wild-field illumination showed that there was no major difference of STAT1 appearance between membrane-associated fraction and cytoplasmic fraction (Supplementary Fig. S4). The only difference was the amount of clusters because many STAT1 proteins were not transported into the nucleus without EGF stimulation. Thus, our work was focused on the cytoplasmic-nuclear region of the cell. Figure 1 showed the distribution of STAT1 in the whole cell with or without EGF stimulation during the interphase. From G1 to G2 phase, we observed that STAT1 accumulated in the nucleus and it was more obvious after EGF stimulation, which was consistent with previous studies that the tyrosine phosphorylated STAT1 could translocate to the nucleus and bind DNA. Previous studies also suggest the existence of STAT1 in the nucleus of unstimulated cells^32^,^33^; herein, our experiment directly proved it.

**Figure 2:**
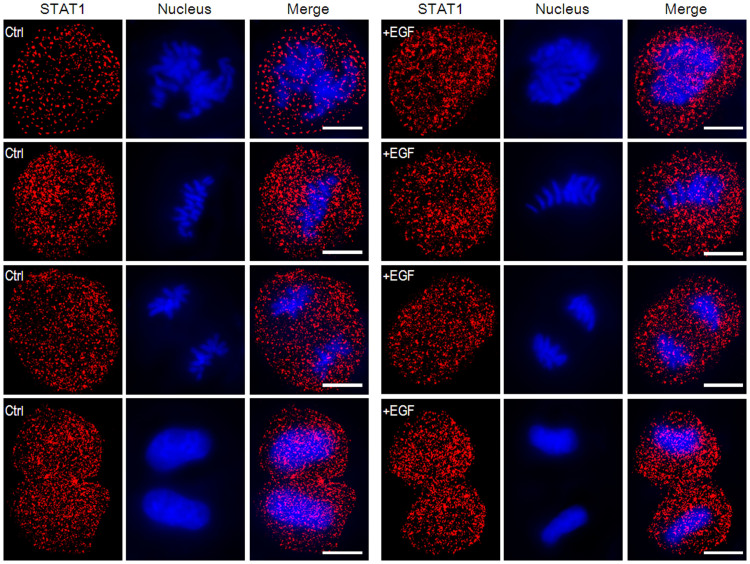
The distribution of STAT1 in the whole cell during the mitosis with or without EGF stimulation. From top to down, Hela cells are classified into four phases according to the nucleus morphology in the mitosis, namely, prophase, metaphase, anaphase and telophase. STAT1 and nucleus were labeled as described above. The left three columns are the control groups and the right ones are the EGF-induced groups. The accumulation of STAT1 during the mitosis is not as evident as in the interphase after EGF stimulation. Scale bars are 10 μm.

Next, we imaged the STAT1 localization in the different phase of the mitotic Hela cells with or without EGF stimulation according to the nucleus morphology (Fig. 2). As the nuclear envelop disappeared and the nuclei broke up at the beginning of mitosis, STAT1 proteins were sporadic in the whole cell with fewer proteins surrounding the chromosomes. Furthermore, there was no significant difference of STAT1 expression level between control groups and EGF-induced groups.

### Characterization of STAT1 cellular localization during the cell cycle

**Figure 3:**
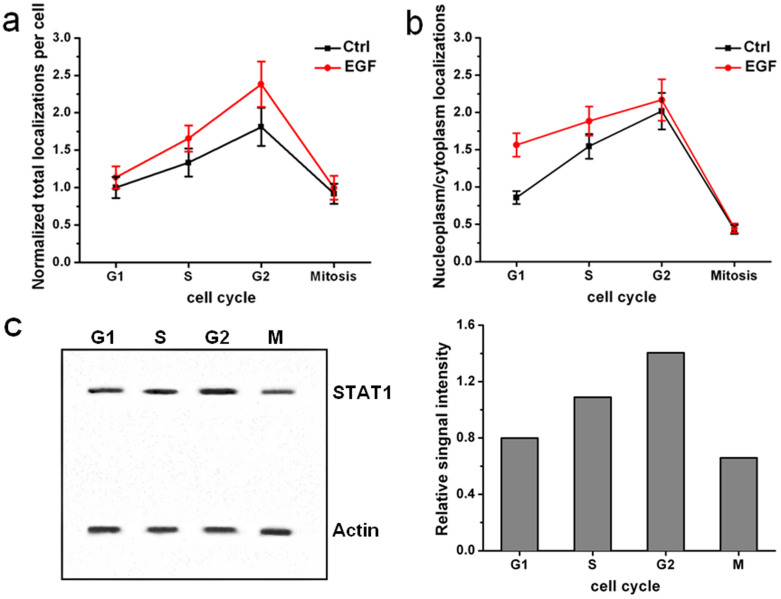
The changes of STAT1 distribution during the cell cycle. (a) Normalized total localizations of individual reconstructed dSTORM images of STAT1 at the different cell-cycle stage with and without EGF stimulation, indicating that the relative quantity of STAT1 proteins increase from G1 to G2 phase and decreased at the mitosis. (b) The ratio of nucleoplasm to cytoplasm localizations of STAT1 proteins during the cell cycle with and without EGF stimulation, showing that STAT1 mainly distributes in the nucleoplasm from G1 to G2 phase after EGF stimulation, and the percentage of STAT1 surrounding the chromosomes decreases significantly in the mitosis. Every control and stimulation group includes 20 cells from four independent experiments. All error bars denote standard deviation (s.d.). (c) Western blot analyses of STAT1 expression level during the cell cycle and quantitation of the data by Image J. The relative signal intensity is calculated after correction for the β-actin loading control.

To quantitatively analyze the STAT1 protein levels and its cellular localization, we calculated the localizations of reconstructed dSTORM images of STAT1 in the whole cell, in the cytoplasm and in the nucleoplasm at the different cell-cycle stage. The average number of localizations for single Alexa647-labeled STAT1 antibody was 29.6 ± 5.4 (s.d.) (100 dyes analyzed, see Methods and Supplementary Fig. S5 for details). Since the number of localizations is almost proportionate to the amount of labeled proteins, we can estimate STAT1 protein levels in different regions of cells by the number of localizations with the statistical method and keeping the experimental conditions as same as possible. Figure 3a showed the normalized total localizations of individual STAT1 images from 20 cells in four independent experiments at the different cell-cycle stage with or without EGF stimulation, which also indicated the changes of STAT1 protein levels. From G1 to G2 phase, the quantity of STAT1 kept growing and reached a maximum at G2 phase. This increasing tendency was more pronounced after EGF stimulation. When it came to the mitosis, the STAT1 levels dropped down and was nearly the same with the levels of G1 phase, whether adding EGF or not. Moreover, western blot analysis also manifested the similar result of dSTORM localizations (Fig. 3c). This may be related to the cell-cycle stage during which the transcription takes place. Generally, DNA transcription carries out during the interphase in which cells produce RNA and proteins required for DNA duplication and cell division. Thus, the STATs activity and expression level is high during the interphase, especially after cytokines stimulation. However, in the mitosis, chromatins became condensed, twined and spiraled to form chromosomes, resulting in ceasing the transcription. Therefore, it may affect the STAT1 expression level and cellular localization, and EGF stimulation does not apparently induce the increase of STAT1 level, either.

As mentioned above, we speculate that the changes of STAT1 amount are associated with physiological behavior of different cell-cycle stage, such as transcription and DNA duplication during the interphase. These activities all take place in the nucleus. To test the hypothesis, we compared the distribution differences of STAT1 in the cytoplasm and nucleoplasm. Figure 3b displayed the ratio of nucleoplasm to cytoplasm localizations of STAT1 proteins during the cell cycle with or without EGF stimulation. Although the nuclear envelop disappeared in the mitosis, we took the chromosome region as ‘nuclear area' to calculate the localizations and the statistical result was the average of each phase of the mitosis. In G1 phase, the ratio was less than one when EGF did not stimulate, demonstrating that STAT1 maintained a prominent accumulation in the cytoplasm. After adding EGF, the ratio rose to more than 1.5, indicating that numerous STAT1 translocated into the nucleus. Transcription and production of proteins for DNA replication usually begin at G1 phase. Therefore, the nuclear accumulation of STAT1 with EGF stimulation during G1 phase confirmed that STAT1 expression level and cellular localization was actually related to the cell-cycle behavior. In subsequent S and G2 phase, the ratio still kept more than 1.5 and grew gradually before reaching a maximum of 2.2 in G2 phase. However, during S and G2 phase, EGF stimulation did not markedly promote the nuclear import of STAT1 as in G1 phase. Maybe the amount of STAT1 in the nucleus gradually saturated during this time and was sufficient to satisfy the requirement of transcription. So even with EGF stimulation, the number of STAT1 in the nucleus did not rise sharply. When cells underwent mitosis, the ratio dropped down to below 0.5, indicating that most of STAT1 distributed in the cytoplasm due to cell division.

### Clustering properties of EGF induced STAT1

**Figure 4:**
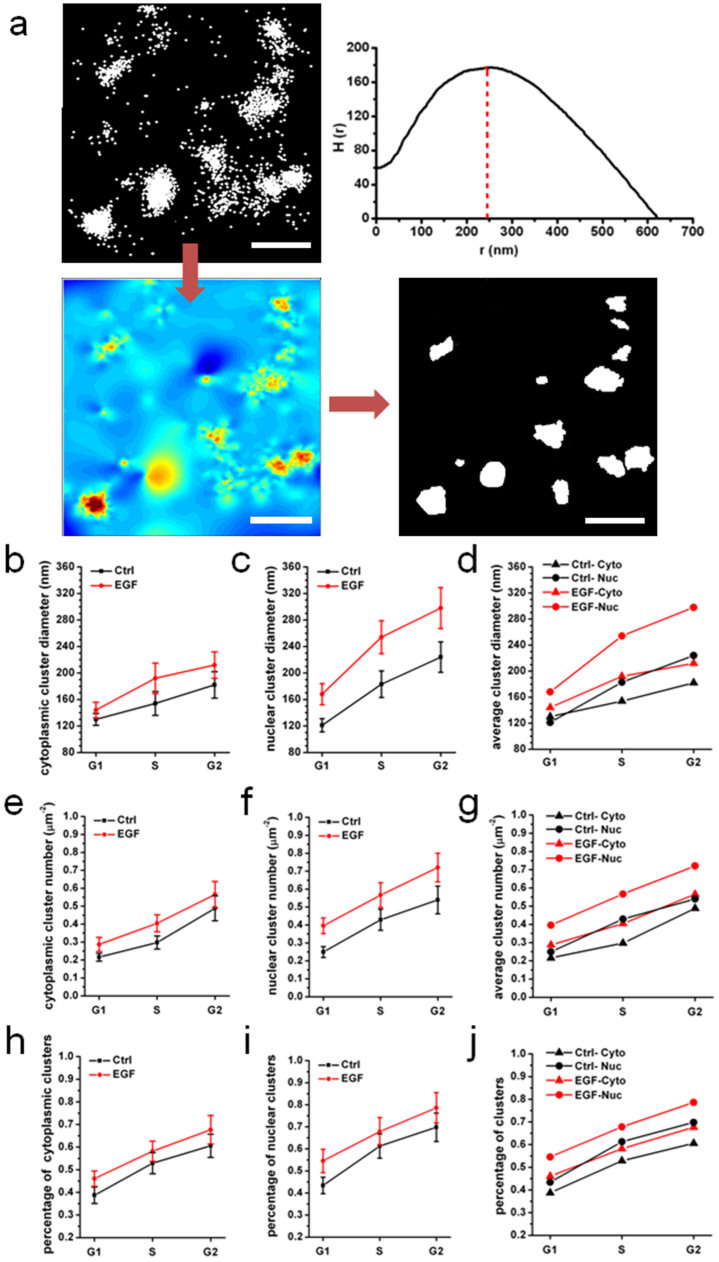
Clustering analysis by Ripley's K function at the different cell-cycle stage. (a) Ripley's K-function analysis of STAT1 protein clustering. The first picture in the left panel is an expanded 2 × 2 μm^2^ region of the dSTORM image of STAT1 in the nucleus of G2-phase cell, showing localization distribution of proteins. Next to it is Ripley's K-function plot showing that the average cluster diameter of maximum clustering scales at 250 nm and clustering range above the level for a random distribution scales up to 625 nm. The second row are the interpolated cluster map based on Ripley's K-function analysis and the binary cluster image generated from the colormap using an appropriate threshold, respectively. The number of clusters, cluster size and other parameters can be extracted from the binary image. Scale bars are 500 nm. (b–d) The distribution of average cluster diameter at the different cell-cycle stage with and without EGF stimulation in the cytoplasm (b), nucleus (c), and in both the cytoplasm and nucleus (d), respectively. (e–g) Comparison of the number of STAT1 clusters per μm^2^ at the different cell-cycle stage with and without EGF stimulation. (h–j) The percentage of STAT1 proteins participating in clusters. Every control and stimulation group includes 20 cells from four independent experiments. All error bars denote standard deviation (s.d.).

We then investigated the distribution pattern of STAT1 in detail in both the cytoplasm and nucleus at the different cell-cycle stage. As known, when STAT1 is activated by tyrosine phosphorylation at amino acid 701, it forms homodimerization or heterodimerization with other STATs through SH2-domain interactions^9^,^34^,^35^, and then the dimers translocate into the nucleus and bind DNA. So we expect that STAT1 may form clusters especially in the nucleoplasm after EGF stimulation. From the magnified dSTORM images of STAT1 (Fig. 1, the last column), we have already observed that some fluorescent localizations assembled tightly, especially in the nucleus during S and G2 phase after EGF stimulation (Fig. 1, the third column indicated by white arrows). Ripley's K-function analysis^36^,^37^ was employed to estimate spatial randomness or aggregation to confirm our presumption again. The K-function (see Methods, Equation 1) is a widely used spatial statistics method and applied to analyze protein distributions in cells in recent years^38^,^39^,^40^. It is often transformed to the H-function (see Methods, Equation 2), where the larger value of H corresponds to the areas of less randomness, and the value of r corresponding to the maximum of H is considered to be the average diameter of clusters in the areas. For instance, we took a 2 × 2 μm^2^ region of the dSTORM image in the nucleus of G2 phase cell to analyze the characteristic of clustering (Fig. 4a). The Ripley's K-function plot revealed that the average clustering diameter was about 250 nm and clustering range above the level for a random distribution scaled up to 625 nm. The color-encoded map of clustering was then created by interpolating a surface plot with L(r) of each points as the z-axis, and the binary cluster map was generated from this color-encoded map by setting a defined L(r) threshold (see Methods). Eventually, the number, size, shape and other parameters of clusters can be obtained from the binary cluster map. We also utilized this method to analyze different regions of STAT1 images in both cytoplasm and nucleoplasm at the different cell-cycle stage with or without EGF stimulation. Supplementary Fig. S6 showed the representative results of Ripley's K-function analyses, indicating that the majority of STAT1 proteins actually formed clusters in the whole cell despite different cluster size. To verify this specific clustering of STAT1, we used Alexa647 goat anti-mouse antibody, a non-STAT1 antibody, as a negative control (Supplementary Fig. S7). The results showed that the protein level was low under non-specific labeling condition and clustering was not as obvious as that with STAT1 labeling. Furthermore, the average cluster diameters were about 30 nm (interphase cells) and 35 nm (mitotic cells) when cells were labeled with non-specific antibodies, which were consistent with the average size of single Alexa647-conjugated antibody molecule (Supplementary Fig. S5). This indicated that non-STAT1 antibodies did not form clusters and further confirmed the facticity of STAT1 clusters.

To visually compare the statistical results of all the plots, we chose the parameter r_ave_ (the average of cluster diameter), as a representative for characterizing these clusters at the different cell-cycle stage. Figure 4b–d showed the distribution of cluster diameter based on Ripley's K-function. We found that the average cluster diameter grew steadily during the interphase, especially from G1 to S phase, both in the cytoplasm (Fig. 4b) and nucleus (Fig. 4c), which may be related to the accumulation of producing proteins and the continuous process of transcription. Moreover, the cluster sizes of EGF-stimulated groups were larger than those of control groups throughout the whole interphase, suggesting that EGF activated STAT1 to form larger clusters. Figure 4d showed the changes of both cytoplasmic and nuclear cluster diameters in each phase with and without EGF stimulation. In G1 phase, cluster size in the cytoplasm was larger than that in the nucleoplasm, which indicated that STAT1 prominently assembled into clusters in the cytoplasm when cells were not induced by EGF. Whereas, after EGF stimulation, the nuclear cluster diameter increased more visibly than the cytoplasmic cluster diameter, which implied that EGF-induced STAT1 clusters were prone to translocate into the nucleus. As for S and G2 phase, cluster size in the nucleoplasm was much larger than that in the cytoplasm, and the difference between them was more impressive after EGF stimulation. This is probably because that in these phases, homodimerized or heterodimerized STAT1 had assembled into larger clusters in the nucleus to participate in transcription, production of proteins and DNA duplication. We also summarized other parameters of Ripley's K-function plots in a list (Supplementary Table S1), such as H(r)_max_ (the degree of clustering) and r_max_ (clustering range), which showed the similar trend.

Based on Ripley's K-function analysis of clustering, we compared the amount of STAT1 clusters in the nucleoplasm and cytoplasm at the different cell-cycle stage with or without EGF stimulation as well (Fig. 4e–g). An increasing number of STAT1 clusters was observed both in the cytoplasm (Fig. 4e) and nucleus (Fig. 4f) from G1 to G2 phase either in control or EGF-induced groups, and EGF-induced groups always had more clusters than control groups. The results also showed that the number of nuclear STAT1 cluster was more than that of cytoplasmic cluster at each stage of the interphase either in control or in EGF-induced groups (Fig. 4g). Furthermore, We found that more and more STAT1 proteins were assembled into clusters as the cell cycle continued, and the percentage of STAT1 in clusters in the nucleoplasm was larger than that in the cytoplasm at each phase either with or without EGF stimulation (Fig. 4h–j). The changes in the STAT1 cluster amount and the percentage of STAT1 participating in clusters were consistent with those in the average cluster diameter and the STAT1 protein distribution. Taken together, our findings support that STAT1 cellular localization and forming clusters are related to cell-cycle behavior; that is, the more active the transcription is, the more STAT1 proteins translocate into the nucleus and form more and larger clusters.

## Discussion

STAT1, as a vital transcription factor responding to inflammatory and growth-factor signals, has attracted widely attentions. Many studies have reported its ability to regulate gene expression and participate in many biologically important signaling systems. As transcription usually happens in the interphase of the cell cycle, we speculate that the cellular localization and nuclear translocation of STAT1 may associate with the timing of transcription. Therefore, we used a super-resolution fluorescence imaging technique-dSTORM to investigate the nucleoplasm and cytoplasm distribution of STAT1 at the different cell-cycle stage. Our work allowed the direct observation of transcription factor at the single molecule level with the resolution of 20–30 nm, and revealed the relationship between STAT1 cellular localization and cell cycle.

Through dSTORM imaging of STAT1 in the whole cell at the different cell-cycle stage with or without EGF stimulation, we found that the distribution and expression level of STAT1 during the cell cycle substantially increased from G1 to G2 phase, and dramatically dropped at the mitosis, and EGF induced the increase of STAT1 amount (Fig. 3a and 3c). Moreover, the nuclear accumulation of STAT1 happened during the interphase and became obvious at G2 phase, while EGF promoted the nuclear import (Fig. 3b). The interphase is a period during which transcription occurs, proteins are produced and DNA is synthesized. Thus, transcription factors are very active during this stage of the cell cycle, which probably leads to the increase of STAT1 expression level. In the mitosis, the main task is that one cell is divided into two daughter cells and chromosomes are transmitted equally to the daughter cell. Transcription is considered to cease during the mitosis, which causes the dramatic reduction in STAT1 amount.

Furthermore, the spatial clustering distribution of STAT1 during the cell cycle by Ripley's K-function analysis uncovers that STAT1 proteins can assemble into different sizes of clusters. The percentage of STAT1 proteins in clusters increased with the cell cycle continuing. In control groups, about 40% of STAT1 proteins formed clusters at G1 phase and this value went up to ~60% at S phase and reached to 70% at G2 phase (Fig. 4h and 4i). Meanwhile, the clustering percentage in the nucleoplasm was larger than that in the cytoplasm at S and G2 phase (Fig. 4j). After EGF stimulation, there was a sharp rise of the clustering percentage and nuclear clustering was more obvious. Besides, the change of the cluster number was also similar with that of the clustering percentage and related to the cell cycle (Fig. 4e–g). Cluster size was much larger at S and G2 phase as well, and the average diameter of clusters in the nucleoplasm was larger than that in the cytoplasm (Fig. 4b–d). Similarly, as transcription and protein production occur during the interphase, these cell-cycle activities promote STAT1 to form more and larger clusters, which may be the oligomerization of STAT1 or the heterodimerization of STAT1 and other STATs. The clustering distribution shortens the distance between STAT proteins, which may promote their interactions and facilitate STAT1 rapid response to signals when required. Nuclear import of STAT1 needs to be tyrosine phosphorylated, and then phosphorylated STAT1 form dimers and bind to specific DNA targets. Thus, upon adding EGF in the cells, STAT1 clusters translocation to the nucleus is very noticeable.

In summary, we find that STAT1 can form various amount and size of clusters in the nucleoplasm and cytoplasm at the different cell-cycle stage, and our results suggest that the cellular localization and distribution pattern of STAT1 is linked to the cell cycle. Transcription and other relevant behavior may enhance the STAT1 expression level and promote the forming of clusters, which may endow STAT1 with stronger capability of transducing signals. Our work lays a foundation for further exploring the mechanisms of cell-cycle regulated STAT1 clustering distribution, and sheds light on the development of new highly targeted means of treatment of STAT-triggered diseases.

## Methods

### Cell culture

Hela cells were cultured in a 5% CO_2_ humidified atmosphere at 37°C in Dulbecco's modified Eagle's medium (Hyclone) supplemented with 10% fetal calf serum (Biochrom AG, Germany), 100 U/ml penicillin and 100 μg/ml streptomycin (Invitrogen). Cells were passaged every two or three days.

### Cell synchronization

After Hela cells in the logarithm period were treated by 2.5 mmol/L thymidine (Sigma-Aldrich) for 16 hours, cells were washed twice by PBS to remove TdR and cultured for 9 hours in DMEM. Then cells were treated by 2.5 mmol/L TdR again for 16 hours, washed twice by PBS and cultured for different time in DMEM continually. At 3.5 h, 8.5 h, 10.5 h and 15 h, cells were collected and stained with PI (Sigma-Aldrich), and then cell cycle was detected by flow cytometry. 70.3% S-phase cells, 84.0% G2-phase cells, 51.2% M-phase cells and 82.1% G1-phase cells were obtained respectively, which showed good cell synchronization (Supplementary Fig. S1). In each experiment, different phases of cells were cultured for fixed time as mentioned above.

### Plasmids and antibodies

Mammalian expression plasmids encoding wild-type human STAT1 fused carboxy-terminally to green fluorescent protein (a gift from Prof. Yue Qin) were used for living cell fluorescence imaging. Alexa647 goat anti-mouse IgG (Invitrogen, A-21236) was used as a non-specific STAT1 antibody. Western blot analyses were done with the following primary antibodies: anti-STAT1α p91 (a mouse monoclonal antibody epitope mapping between amino acids 613–739 of STAT1α p91 of human origin, from Santa Cruz, C-111) and anti-β-Actin mouse monoclonal antibody (Transgen Biotech, #I10813). Peroxidase-conjugated goat anti-mouse IgG (Proteintech, SA00001-1) was used as the secondary antibody.

### Cytokine treatment, cell fixation and permeabilization

Cells were seeded onto pre-cleaned standard microscope slides. Activation of STAT1 was done by treating cells with 50 ng/ml EGF (Peprotech) for 1 hour before collecting different phases of cells. Then different phases of cells were fixed in 4% paraformaldehyde (PFA, Fisher) for 15 min at the room temperature at each time point, washed three times by PBS, treated with 0.1% Triton X-100 (Roche) for 20 min or methanol (MeOH) for 1 min at −20°C, and washed three times by PBS again. Finally, cells were stored in PBS at 4°C in dark for use.

### Staining and sample preparation

At first, anti-STAT1α p91 antibodies were labeled with Alexa647 (Invitrogen) in an appropriate concentration. 100 μl STAT1 (100 μg/ml) antibodies were stained by 2 μl Alexa647 (1 mg/ml, dissolved in DMSO) and shook for 2 hours in dark at room temperature. After reacting completely, the solution was filtered out by gel filtration using illustra NAP-5 columns (GE Healthcare) to remove excess dyes. The A_650_ and A_280_ was read to determine that the labeling ratio of Alexa647 and antibody was 0.7~1 dye/protein by absorption spectroscopy assay. Appropriate fractions were pooled for use.

Prepared cell samples were blocked by incubating in 1% BSA for 30 min. After washing out the blocking buffer by PBS for three times, cells were stained with 50 μl Alexa647- conjugated STAT1 antibodies, as described above, for 40 min in dark at room temperature. Then cells were incubated with 1:500 dilution of Hoechst33342 (Sigma-Aldrich) for 10 min, and washed out the staining solutions for three times with PBS again.

Prior to imaging, cell samples were immersed in a STORM imaging buffer, containing Tris (50 mM, pH 8.0), NaCl (10 mM), glucose (10% w/v), β -mercaptoethanol (1% v/v; Sigma–Aldrich), glucose oxidase (500 μg/ml; Sigma–Aldrich), and catalase (40 μg/ml; Sigma–Aldrich) in PBS, and mounted on pre-cleaned glass slides.

### Microscopy setup and image acquisition

STORM imaging was performed on a Nikon Ti-E microscope with a 100 × 1.49 NA TIRF lens (Nikon, Japan). Excitation was provided by diode-pumped, solid-state lasers emitting at 640 nm. The imaging laser had an output power in the 80 mW range, corresponding to a total irradiance of approximately 30 mW at the sample. Diffraction-limited images were acquired prior to dSTORM in wide-field illumination. The sample was firstly illuminated by 405 nm laser to obtain a single image of the nucleus, and then was excited by 640 nm laser to acquire the Alexa647 signal for imaging STAT1. 5,000–10,000 images were captured for each cell with 40 ms internal per frame for the reconstruction of the super-resolution image. The system was equipped with one dichromic filter which reflects both 405 nm and 647 nm laser (Chroma Technology). In order to maximize spatial registration of the sequentially collected fluorescence images from Alexa647 and Hoechst33342, one dual-band emission filter which collects fluorescence at 420–480 nm and at 655–870 nm (Chroma Technology) was used to avoid switching fluorescence cubes. Fluorescent signals were collected via a cooled EMCCD camera (Photometrics, Cascade II). Obtaining one single dSTORM image usually took less than 7 minutes. During this short acquisition time, the z-drift was eliminated by a focus lock, and TetraSpeck microspheres (Invitrogen) were embedded as fiducial markers to correct the x-y drift of the sample and characterize the optical registration for dual color imaging.

### Image reconstruction

For imaging data analyses, we used a freely available plug-in for Image J named quickPALM^41^ to analyze raw images^42^. Image TIFF stacks were first preprocessed via background subtraction. For each frame, points corresponding to single photoemission events were identified with a minimum SNR of 2–4. Then fluorescence peaks were identified in each frame and fitted a least-squares fit with an elliptical Gaussian function. Individual least-squares fit estimates were performed by a threshold of the peak height and the peak widths in the two lateral dimensions. After rejecting the poor fit and asymmetric point spread functions (PSFs), the coordinates of detected molecules were determined by the centers of gravity of their PSFs. STORM images were reconstructed as a density map using the precise localization data of single fluorescent molecules. The reconstructed dSTORM image of STAT1 within the cell was overlapped with the single fluorescent image of the nucleus via Image-Pro Plus 6.0 (Media Cybernetics, Inc.) to obtain the two-channel merging image.

To detect the resolution in our set-up instrument, single molecule localization precision was measured^39^. Adequately diluted Alexa647 conjugated anti-STAT1 antibodies (about 10 nM) were added onto the prepared cell surface, incubated for 40 min, and imaged. From repetitively switching fluorophores, the localization precision was determined to be 30 nm for Alexa647-STAT1 antibody by measuring the full-width at half-maximum (FWHM) of the localization distribution of individual fluorophores (Supplementary Fig. S2). Meanwhile, the average number of localized spots in a single Alexa647-labeled STAT1 antibody was calculated, which was 29.6 ± 5.4 (s.d.) (100 dyes analyzed).

### Cluster analysis

To analyze the distribution of STAT1 in the whole cell, Ripley's K-function^36^,^37^ was applied to characterize STAT1 clustering based on the localization data established as described above. The examined region of 2 × 2 μm^2^ in the reconstructed dSTORM image was selected. Ripley's K-function was then calculated as:
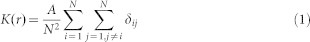
Where *A* is the image area, *N* is the number of total localizations in the area, *r* is the spatial scale (radius) for the K-function calculation and *δ_ij_* is the distance between points the *i*-th and the *j*-th localizations. Here, if *δ_ij_* is less than *r*, the value will be one, otherwise *δ_ij_* = 0. This essentially counts the number of other points encircled by concentric rings centered on each point. The linear transformation of *K(r)*, namely H-function, was used to interpret the spatial randomness:
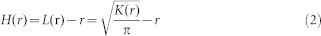
The amplitude of *H(r)* would be zero for particles with a random distribution, and positive for clustering particles. Edge-effects were negated by weighting edge points and cropping image edges after the calculation. The values of *L(r)* generated by each particle were used to produce a cluster map by interpolating a surface plot with *L(r)* as the z-axis. Then a binary cluster map was generated through a *L(r)* threshold. If the percentage of points satisfying *H(r)* ≤ 0 was 30%, the *L(r)* threshold was set at 30% of the maximum *L(r)* value from the plot. Finally the information of clustering could be extracted from the binary map, such as the number and the size of clusters. All calculations and image processing were performed in Matlab.

## Author Contributions

H.W. supervised the project. J.G. performed the experiments and the analysis. F.W. and Y.L. detected the cell cycle by flow cytometry. M.C., H.X. and J.J. coordinated the study. J.G. and H.W. wrote the manuscript. All authors discussed the results and commented on the manuscript.

## Supplementary Material

Supplementary Information Revealing the cellular localization of STAT1 during the cell cycle by super-resolution imaging

## References

[b1] Darnell J. E., Kerr I. M. & Stark G. R. JAK-STAT pathways and transcriptional activation in response to IFNs and other extracellular signaling proteins. Science 264, 1415–1421 (1994). 8197455 10.1126/science.8197455

[b2] Karin M. & Hunter T. Transcriptional control by protein phosphorylation: signal transmission from the cell surface to the nucleus. Curr. Biol. 5, 747–757 (1995). 7583121 10.1016/s0960-9822(95)00151-5

[b3] Darnell J. E. STATs and gene regulation. Science 277, 1630–1635 (1997). 9287210 10.1126/science.277.5332.1630

[b4] Horvath C. M. STAT proteins and transcriptional responses to extracellular signals. Trends Biochem. Sci. 25, 496–502 (2000). 11050435 10.1016/s0968-0004(00)01624-8

[b5] Bromberg J. & Darnell J. E. Jr The role of STATs in transcriptional control and their impact on cellular function. Oncogene 19, 2468–2473 (2000). 10851045 10.1038/sj.onc.1203476

[b6] Levy D. E. & Darnell J. Stats: transcriptional control and biological impact. Nat. Rev. Mol. Cell Biol. 3, 651–662 (2002). 12209125 10.1038/nrm909

[b7] Haura E. B., Turkson J. & Jove R. Mechanisms of disease: Insights into the emerging role of signal transducers and activators of transcription in cancer. Nat. Clin. Pract. Oncol. 2, 315–324 (2005). 16264989 10.1038/ncponc0195

[b8] Ihle J. N. The Stat family in cytokine signaling. Curr. Opin. Cell Biol. 13, 211–217 (2001). 11248555 10.1016/s0955-0674(00)00199-x

[b9] Schindler C., Shuai K., Prezioso V. R. & Darnell J. E. Interferon-dependent tyrosine phosphorylation of a latent cytoplasmic transcription factor. Science 257, 809–813 (1992). 1496401 10.1126/science.1496401

[b10] Shuai K., Schindler C., Prezioso V. R. & Darnell J. E. Activation of transcription by IFN-gamma - tyrosine phosphorylation of a 91-KD DNA-binding protein. Science 258, 1808–1812 (1992). 1281555 10.1126/science.1281555

[b11] David M. *et al.* STAT activation by epidermal growth factor (EGF) and amphiregulin - Requirement for the EGF receptor kinase but not for tyrosine phosphorylation sites or JAK1. J. Biol. Chem. 271, 9185–9188 (1996). 8621573 10.1074/jbc.271.16.9185

[b12] Olayioye M. A., Beuvink I., Horsch K., Daly J. M. & Hynes N. E. ErbB receptor-induced activation of Stat transcription factors is mediated by Src tyrosine kinases. J. Biol. Chem. 274, 17209–17218 (1999). 10358079 10.1074/jbc.274.24.17209

[b13] Vignais M.-L., Sadowski H. B., Watling D., Rogers N. C. & Gilman M. Platelet-derived growth factor induces phosphorylation of multiple JAK family kinases and STAT proteins. Mol. Cell. Biol. 16, 1759–1769 (1996). 8657151 10.1128/mcb.16.4.1759 PMC231162

[b14] Chin Y. E. *et al.* Cell growth arrest and induction of cyclin-dependent kinase inhibitor p21(WAF1/CIP1) mediated by STAT1. Science 272, 719–722 (1996). 8614832 10.1126/science.272.5262.719

[b15] Dimco G., Knight R. A., Latchman D. S. & Stephanou A. STAT1 interacts directly with cyclin D1/Cdk4 and mediates cell cycle arrest. Cell Cycle 9, 4638–4649 (2010). 21084836 10.4161/cc.9.23.13955

[b16] Turkson J. & Jove R. STAT proteins: novel molecular targets for cancer drug discovery. Oncogene 19, 6613–6626 (2000). 11426647 10.1038/sj.onc.1204086

[b17] Koster M. & Hauser H. Dynamic redistribution of STAT1 protein in IFN signaling visualized by GFP fusion proteins. Eur. J. Biochem. 260, 137–144 (1999). 10091593 10.1046/j.1432-1327.1999.00149.x

[b18] Begitt A., Meyer T., van Rossum M. & Vinkemeier U. Nucleocytoplasmic translocation of Stat1 is regulated by a leucine-rich export signal in the coiled-coil domain. PNAS 97, 10418–10423 (2000). 10973496 10.1073/pnas.190318397 PMC27039

[b19] Subramaniam P. S., Larkin J., Mujtaba M. G., Walter M. R. & Johnson H. M. The COOH-terminal nuclear localization sequence of interferon gamma regulates STAT1 alpha nuclear translocation at an intracellular site. J. Cell Sci. 113, 2771–2781 (2000). 10893192 10.1242/jcs.113.15.2771

[b20] Lillemeier B. F., Koster M. & Kerr I. M. STAT1 from the cell membrane to the DNA. EMBO J. 20, 2508–2517 (2001). 11350940 10.1093/emboj/20.10.2508 PMC125461

[b21] Huang B., Bates M. & Zhuang X. Super-resolution fluorescence microscopy. Annu. Rev. Biochem. 78, 993–1016 (2009). 19489737 10.1146/annurev.biochem.77.061906.092014 PMC2835776

[b22] Schermelleh L., Heintzmann R. & Leonhardt H. A guide to super-resolution fluorescence microscopy. J. Cell Biol. 190, 165–175 (2010). 20643879 10.1083/jcb.201002018 PMC2918923

[b23] Allen J. R., Ross S. T. & Davidson M. W. Single molecule localization microscopy for superresolution. J. Opt. 15, 094001 (2013). 10.1039/c3cp53719f 24084850

[b24] Rust M. J., Bates M. & Zhuang X. Sub-diffraction-limit imaging by stochastic optical reconstruction microscopy (STORM). Nat. Meth. 3, 793–795 (2006). 10.1038/nmeth929 PMC2700296 16896339

[b25] Heilemann M. *et al.* Subdiffraction-resolution fluorescence imaging with conventional fluorescent probes. Angew. Chem. Int. Edit. 47, 6172–6176 (2008). 10.1002/anie.200802376 18646237

[b26] Betzig E. *et al.* Imaging intracellular fluorescent proteins at nanometer resolution. Science 313, 1642–1645 (2006). 16902090 10.1126/science.1127344

[b27] Klenerman D., Korchev Y. E. & Davis S. J. Imaging and characterisation of the surface of live cells. Curr. Opin. Chem. Biol. 15, 696–703 (2011). 21536476 10.1016/j.cbpa.2011.04.001

[b28] Sengupta P. *et al.* Probing protein heterogeneity in the plasma membrane using PALM and pair correlation analysis. Nat. Meth. 8, 969–975 (2011). 10.1038/nmeth.1704 PMC3400087 21926998

[b29] Gunzenhaeuser J., Olivier N., Pengo T. & Manley S. Quantitative super-resolution imaging reveals protein stoichiometry and nanoscale morphology of assembling HIV-Gag virions. Nano Lett. 12, 4705–4710 (2012). 22906128 10.1021/nl3021076

[b30] Schnell U., Dijk F., Sjollema K. A. & Giepmans B. N. G. Immunolabeling artifacts and the need for live-cell imaging. Nat. Meth. 9, 152–158 (2012). 10.1038/nmeth.1855 22290187

[b31] Landgraf D., Okumus B., Chien P., Baker T. A. & Paulsson J. Segregation of molecules at cell division reveals native protein localization. Nat. Meth. 9, 480–U498 (2012). 10.1038/nmeth.1955 PMC3779060 22484850

[b32] Meyer T., Begitt A., Lodige I., van Rossum M. & Vinkemeier U. Constitutive and IFN-gamma-induced nuclear import of STAT1 proceed through independent pathways. EMBO J. 21, 344–354 (2002). 11823427 10.1093/emboj/21.3.344 PMC125830

[b33] Meyer T., Gavenis K. & Vinkemeier U. Cell type-specific and tyrosine phosphorylation-independent nuclear presence of STAT1 and STAT3. Exp. Cell Res. 272, 45–55 (2002). 11740864 10.1006/excr.2001.5405

[b34] Fu X. Y. A transcription factor with SH2 and SH3 domains is directly activated by an interferon-alpha-induced cytoplasmic protein trosine kinase(s). Cell 70, 323–335 (1992). 1638633 10.1016/0092-8674(92)90106-m

[b35] Gutch M. J., Daly C. & Reich N. C. Tyrosine phosphorylation is required for activation of an alpha-interferon-stimulated transcription factor. PNAS 89, 11411–11415 (1992). 1280824 10.1073/pnas.89.23.11411 PMC50560

[b36] Ripley B. D. Tests of randomness for spatial point patterns. J. Roy. Stat. Soc. B-Meth. 41, 368–374 (1979).

[b37] Owen D. M. *et al.* PALM imaging and cluster analysis of protein heterogeneity at the cell surface. J. Biophotonics 3, 446–454 (2008). 20148419 10.1002/jbio.200900089

[b38] Aaron J. S., Carson B. D. & Timlin J. A. Characterization of Differential Toll-like Receptor Responses below the Optical Diffraction Limit. Small 8, 3041–3049 (2012). 22807232 10.1002/smll.201200106 PMC3613986

[b39] Wu J. *et al.* High-efficiency localization of Na+-K+ ATPases on the cytoplasmic side by direct stochastic optical reconstruction microscopy. Nanoscale 5, 11582–11586 (2013). 24113832 10.1039/c3nr03665k

[b40] Lehmann M. *et al.* Quantitative Multicolor Super-Resolution Microscopy Reveals Tetherin HIV-1 Interaction. Plos Pathog. 7, e1002456 (2011). 22194693 10.1371/journal.ppat.1002456 PMC3240612

[b41] Henriques R. *et al.* QuickPALM: 3D real-time photoactivation nanoscopy image processing in ImageJ. Nat. Meth. 7, 339–340 (2010). 10.1038/nmeth0510-339 20431545

[b42] Wolter S. *et al.* Real-time computation of subdiffraction-resolution fluorescence images. J. Microsc-Oxford 237, 12–22 (2009). 10.1111/j.1365-2818.2009.03287.x 20055915

