# Supplementary material for: Revealing the cellular localization of STAT1 during the cell cycle by super-resolution imaging
Source: Sci Rep. 2015 Mar 12;5:9045. doi: 10.1038/srep09045 (PMC4356954; doi:10.1038/srep09045)
Supplement: Supplementary Information — Revealing the cellular localization of STAT1 during the cell cycle by super-resolution imaging [file srep09045-s1.pdf]

SUPPLYMENTARY INFORMATION

Revealing the cellular localization of STAT1 during the cell cycle by super-resolution imaging

Jing Gao[1,2], Feng Wang[1], Yanhou Liu[3], Mingjun Cai[1], Haijiao Xu[1], Junguang Jiang[1], Hongda Wang[1,*]

[1]State Key Laboratory of Electroanalytical Chemistry, Changchun Institute of Applied Chemistry, Chinese Academy of Sciences, Changchun, China, [2]Graduate University of Chinese Academy of Sciences, Beijing, China, [3]Institute of Immunology, The First Bethune Hospital Academy of Translational Medicine, Jilin University, Changchun, China

*Correspondence to hdwang@ciac.ac.cn

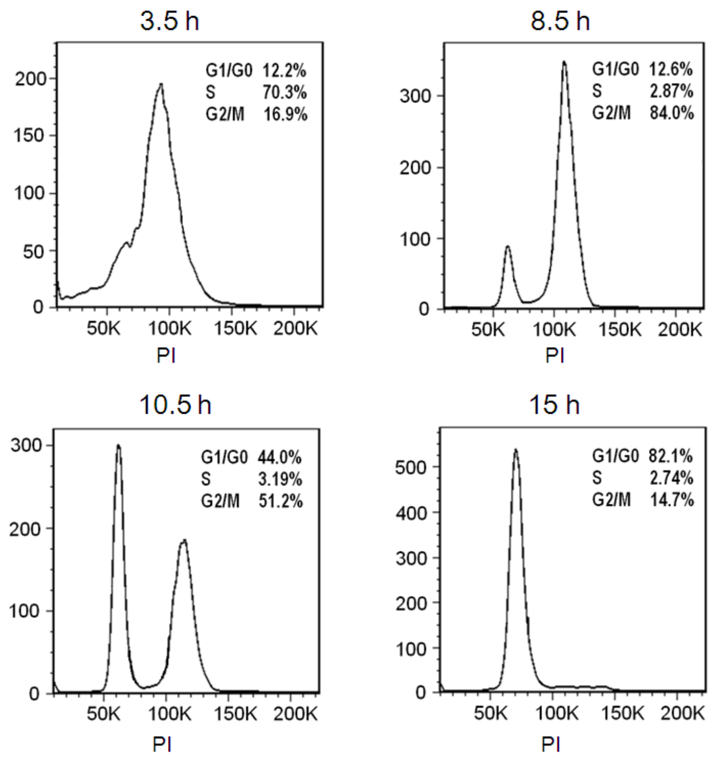

Figure S1. Cell cycle analysis by flow cytometry.

Hela cells were synchronized with TdR treatment and collected at the indicated time points. After PI staining, the DNA content was analyzed by means of flow cytometry. G0/G1, G2/M, and S indicate cell phase. The percentage of cells in each phase of the cell cycle is presented. Data shown are the representative of three independent experiments.

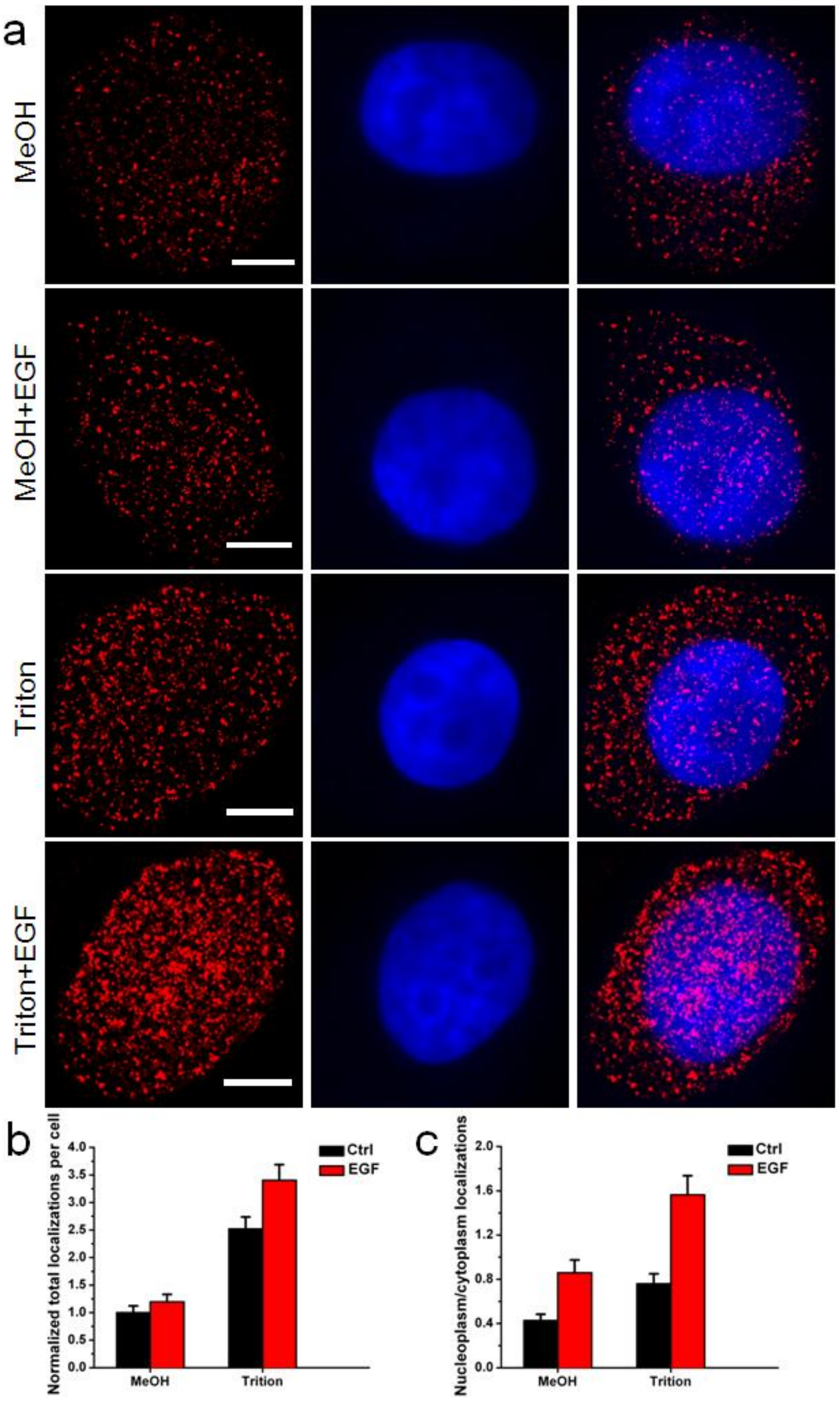

Figure S2. Optimization of fixation and permeabilization conditions.

(a) dSTORM images of Hela cells with or without EGF stimulation after fixation with

4% PFA, permeabilization with 0.1% Triton X-100 (Triton) for 20 min at room temperature or methanol (MeOH) for 1 min at -20℃, and staining with anti-STAT1 antibodies and Hoechst33342. Scale bars are 10 μm. (b) Normalized total localizations of STAT1 dSTORM images of Hela cells permeated with Triton X-100 or MeOH as in (a) before and after EGF stimulation. (c) The ratio of nucleoplasm to cytoplasm localizations of STAT1 with different permeabilization methods before and after EGF stimulation. Every control and stimulation group includes 15 cells from three independent experiments. All error bars denote standard deviation (s.d.).

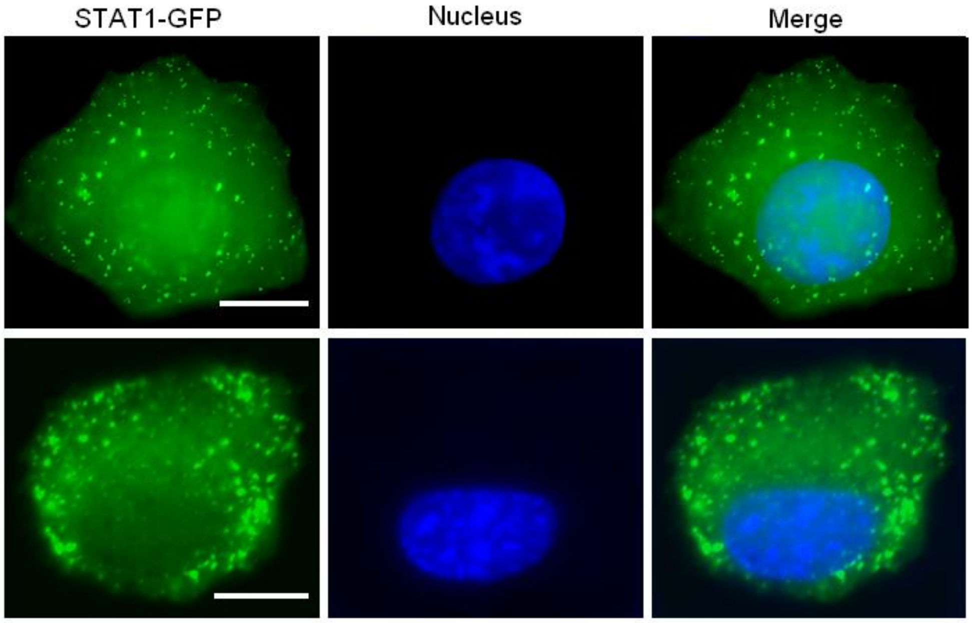

Figure S3. STAT1 fluorescence imaging in living cells.

Hela cells were transfected with plasmids encoding GFP-fused STAT1 (STAT1-GFP) and stained with Hoechst33342 before fluorescence imaging. Two representative fluorescence images of STAT1 proteins and the nuclei in Hela cells during the interphase are shown. The merged images are also displayed. Scale bars are 10 μm.

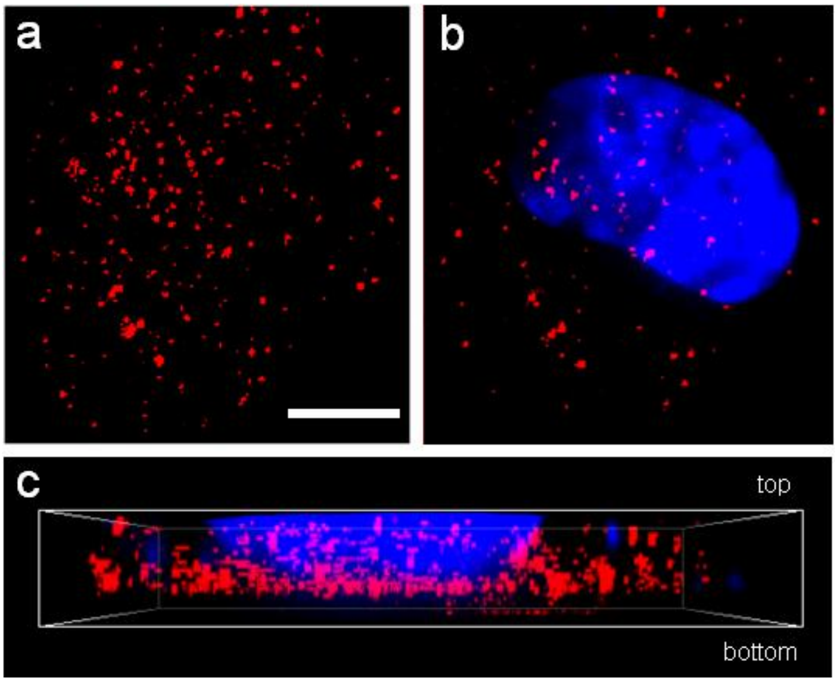

Figure S4. Three dimensional fluorescence imaging of STAT1 in Hela cells. (a) The fluorescence image of STAT1 in the juxtamembrane by TIRF illumination. Hela cells were fixed, permeated and stained with Alexa647-conjugated STAT1 antibodies and Hoechst33342 before imaging. Scale bar is 10 μm. (b) The fluorescence image of STAT1 and the nucleus in the cytoplasm of the same cell by wide-field illumination. The image is located approximately 3 μm above the bottom of the cell. (c) The 3D image of STAT1 and the nucleus of the same cell constructed by successive z-stacks spaced by 200 nm from the bottom to the top of the cell. The total recorded z-axis distance is 5 μm.

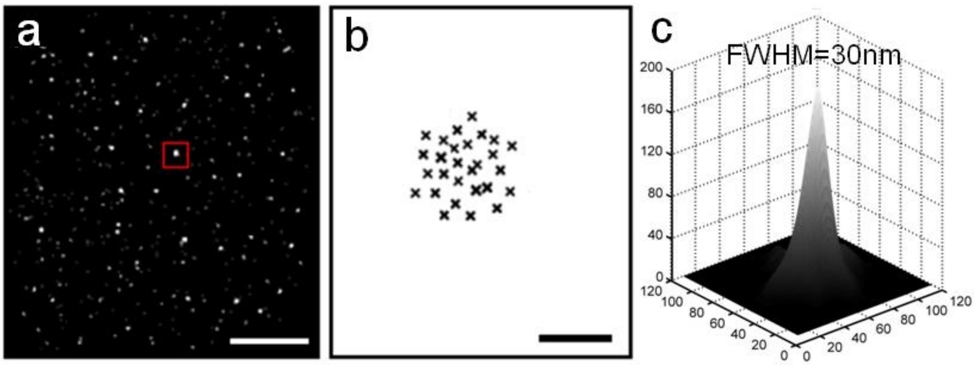

Figure S5. Quantitative analysis of the localization precision of single Alexa647 conjugated STAT1 antibody in Hela cells.

(a) The reconstructed dSTORM image of adequately diluted Alexa647-SATA1 antibody molecules (≈10nM) in Hela cells. (b) Repetitive localizations of one Alexa647-STAT1 antibody labeled in (a) are represented after sample drift correction. (c) Aligned two-dimensional distribution of localizations from 100 fluorescent molecules obtains a mean spatial resolution of 30 nm. Scale bars are 1 μm for (a), and 20 nm for (b).

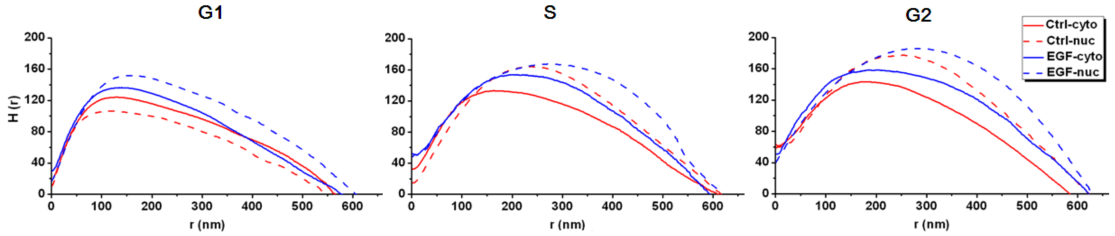

Figure S6. Ripley's K-function plot of STAT1 cluster analysis.

Ripley's K function was processed in the 2×2 μm^2 region of reconstructed dSTORM images of STAT1 in both cytoplasm (Cyto) and nucleoplasm (Nuc) at the different cell-cycle stage with and without EGF stimulation. Plots shown are the representatives of 60 analyzed regions from 20 cells.

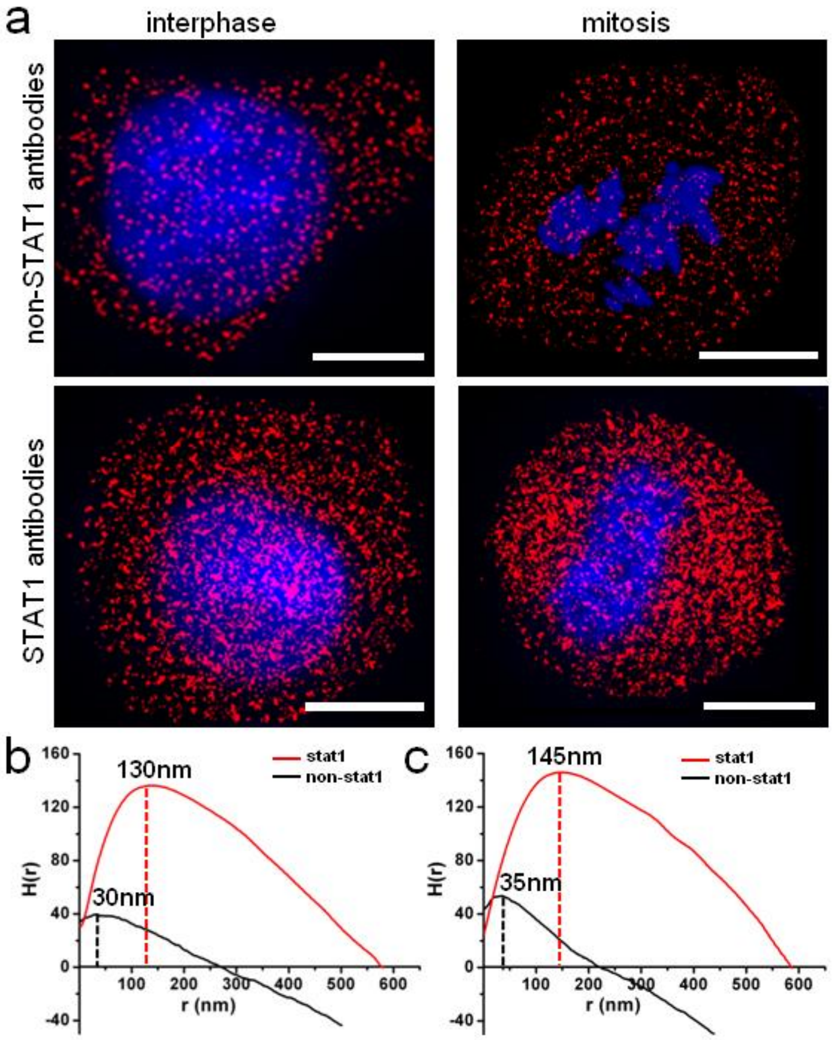

Figure S7. Comparison of non-specific and specific STAT1 labeling.

(a) Hela cells during the interphase and mitosis were stained with Alexa647 goat anti-mouse antibodies (non-STAT1 antibodies) or Alexa647-conjugated STAT1 antibodies (STAT1 antibodies) and Hoechst33342 after fixation and permeabilization. The merged images of 640 nm and 405 nm channels are shown. Scale bars are 10 μm. (b-c) Ripley's K-function analysis of protein clustering under non-specific (black plots) and specific STAT1 (red plots) labeling conditions. The 2×2 μm^2 regions of the dSTORM images in the cytoplasm of the interphase cell (b) and mitotic cell (c) are analyzed. The average cluster diameters of maximum clustering are 30 nm and 35 nm for non-specific STAT1 labeling, and 130 nm and 145 nm for specific STAT1 labeling.

Cell-cycle stage	treatment	cytoplasm			nucleoplasm		
		r_{ave} (nm)	H (r)$_{max}$	r_{max} (nm)	r_{ave} (nm)	H (r)$_{max}$	r_{max} (nm)
G1	Ctrl	130±9	124±12	565±42	121±10	106±10	544±38
	+EGF	144±12	136±10	580±46	168±16	153±13	607±41
S	Ctrl	154±18	134±13	612±54	183±20	165±14	616±39
	+EGF	192±23	154±14	593±37	254±25	169±17	621±44
G2	Ctrl	182±20	143±12	586±31	224±23	177±18	625±51
	+EGF	212±20	158±16	624±45	298±31	186±16	630±40

Table S1. Analysis of STAT1 clustering by Ripley's K function.

Ripley's K function was calculated in the 2×2 μm^2 region of reconstructed dSTORM images of STAT1 at the different cell-cycle stage with and without EGF stimulation. Every control and stimulation group included 20 cells from four independent experiments, and each cell was chosen three regions in both cytoplasm and nucleoplasm. The values of every parameter are the average of the total data including the standard deviation (mean±s.d.). $H(r)_{max}$ is the vertex of the curve, which reveals the localization density within the clusters; r_{ave} is the r value corresponding to the maximum of $H(r)$, which represents the average cluster diameter of maximum clustering; and r_{max} is the intercept of the curve at the horizontal axis, which means the maximal clustering range above the level for a random distribution.
